# At the Chicago Medical Society

**Published:** 1884-06

**Authors:** 


### Society Reports.

### AT THE CHICAGO MEDICAL SOCIETY.

Regular meeting of March 17, 1884.

The following important topics were discussed, regarding operative surgery pertaining to the nervous system, namely:

I.–Suture of Nerves. II.–Elongation of Nerves. III.–Exploration of the Brain by the Hollow Needle. The above selections appeared in a paper, prepared by Dr. Roswell Park, of Buffalo, N.Y., wherein he has deduced a number of interesting conclusions, a synopsis of which is hereby appended, furnished by the Secretary, who read the paper for the author (unavoidably absent).

We read* that Gillaume de Salicet, Lisfranc, Guy de Chauliac, and others, recommended the suture of divided nerves. But when we remember that nerves and tendons were often confounded in those days, this interesting item loses part of its importance. In 1776, Cruikshank demonstrated the possibility of cicatrization of nerves; after him Fontana proved this cicatricial substance to consist more or less of true nerve tissue, and recommended its union by suture, when divided, a procedure further advised by Dupuytren.

But the first to actually practice it as a physiological experiment on human patients were Baudens, Laugier, and Nelaton; the two latter having each within a single week, in 1864, a case which each for his own part reported.† It happened very curiously that in each of these cases it was claimed that sensibility and motility had returned within 24 hours after operation. This

* International Encyclopedia of Surgery, III, 621.

† Comptes rendus, 1864. No. 25.

seemed incredible, and Richet* cleared up the matter by reporting a case. This author claimed, and properly, that a similar state of affairs had obtained in those cases in which Laugier and Nelaton had claimed such surprising results, and that they had not accurately studied their cases before operating. Since that time the subject has been studied experimentally and clinically, until now the indications to the surgeon are perfectly clear. In the literature on this subject may be found among others the views of Arloing and Tripier, Langerfeldt, Lemke, Kraussold, Page, Brown-Sequard, Tillmanns, Nicaise, and Holmes. The exact number of all cases recorded to date, as nearly as the writer can estimate, is thirty, nearly all of which have been successful. To these, he would add two more never yet reported, some features of the operation.

CASE I.—*Rupture of the Sciatic Nerve—Immediate Union—Restoration of Function.* H. H., æt. 13. In 1881 the surgeon excised the hip joint of this patient (who was then 11 years of age), for long-standing and extensive disease. He made a good recovery from a severe operation. In time sinuses burrowed in the region of the buttocks, along with other evidence of new and extensive disease, and Feb. 9, 1883, radical operative measures to remove the disease were again undertaken, the patient being admitted to the Michael Reese Hospital, Chicago. He was assisted by Drs. Lackner, Tracy, and McAuliffe, and proceeded first to explore the parts. He found caries of the tuberosity and other parts of the ischium, sinuses leading inside the pelvic cavity and around the rectum. In brief, aggravated tuberculous disease of the bony pelvis and contents was present. In order to secure access to the parts, and to permit the necessary freedom of manipulation, a flap composed of the buttocks had to be laid up on that side, owing to the cicatrization following the previous operation and the present local disease and induration. The sheath of the sciatic nerve had contracted firm adhesions to the overlying parts. The nerve was unmistakable, but the tissue was in the way. So one of the gentlemen present undertook to hold up the flap with a powerful retractor, while the operator dissected the tissues from the nerve. Being short of sufficient assistance,

** Union Med.*, 1867, p. 444; *Gaz. des Hôpitaux* 1867, p. 519 *et seq.*

the patient inadvertently was permitted to so far recover from his chloroform that he gave a violent wrench and turned partially around, when it was discovered that the nerve had been torn completely through; the ends, while not exactly ragged, being anything but smooth. The ends were dissected free, and the operation proceeded with as had been originally contemplated, *i.e.*, by resection of a good portion of the ischium, scraping out of the sinuses leading into the pelvis, inserting drainage tubes, etc., after which the nerve-ends were united, first snipping them off evenly with scissors, and then sutured as accurately as possible by two catgut threads passed entirely through the nerve-trunk and about a quarter of an inch from the line of section. After this the soft parts were closed as usual. Proper provision was made for drainage, and a Lister dressing applied, the whole having been done under the carbolic spray.

For about a week there was absolute paralysis of sensation and motion throughout the region supplied by the great sciatic. Thirteen days after operating there was but a small area of integument near the external malleolus which was not sensitive as usual, while he could move his leg as well as before the operation, and judging by his progress, regeneration was practically complete in a day or two more.

CASE II. *Division of Radial Nerve and Certain Tendons; Suture of Same after Two Weeks—Restoration of Function.* C. D. G., aged 16. April 1, 1883, patient cut his right wrist with a cheese knife in a way that severed the radial nerve and artery, and the extensor ossis metacarpi pollicis and extensor primi internodii pollicis. The physician who saw him at the time of the accident contented himself with checking the hæmorrhage. At the Reese Hospital, Chicago, on the 16th, the following condition was noted: Hand dropped to the ulnar side, with sensibility of those parts supplied by the radial nerve, extremely impaired, and, indeed, in the central portion of that area practically lost.

After anæsthetizing him and dissecting down upon the parts, found the tendon ends retracted within their sheaths, so as to be an inch apart; opened the sheaths and carefully united the tendons by catgut suture, and their sheaths over them by interrupted sutures of the same material. Then the ends of the radial nerve were found, and freshened and brought together by a single catgut thread. Over all, iodoform dressings. By May 5, sensation was restored to the areas supplied by the radial nerve; by the 25th, it was evident that the tendons had firmly re-united, and, except as limited by partial adhesions in their sheaths, were capable of re-assuming their functions. In course of time these adhesions became stretched, and the hand became very nearly as useful as before.

*Commentary.* Suture of nerves is known as *direct* when the suture material is passed directly through the nerve trunk, and as *indirect* when only the nerve sheath is included; the former is usually preferable. There is fair prospect of success, even months after the original injury, as witness the case in which Wheelhouse* united the sciatic fully nine months after the accident which severed it, the patient in four months more being able to do all manner of hard work; while Jessop, according to Tillmanns,† has recently operated *nine years* after the injury and with fair success. Nevertheless, the sooner it is done, the better, and the quicker will be the result.

If on dissection the nerve ends prove to be clean cut (in recent cases) they need no further attention before uniting them, and the ordinary full curved surgical needle will suffice for the operation, taking care to insert and pass it flat-wise between the bundle of nerve fibers until the broader cutting part is through, and then to turn the needle half round and let the carbolized cat-gut slip through the little slit. It may be necessary to introduce two or three sutures, especially in the larger trunks. Avoid tension of the limb. The part must be dressed accordingly, and immobilized. Observe full antiseptic measures. Evidently primary union is only such in appearance. It is cicatricial, not physiological. *Immediate suture does not prevent degeneration, but simply permits regeneration to take place* much more rapidly than it otherwise would. But *immediate union of divided nerve tissue does not occur.* Letiévant has suggested autoplastic operations

---

* *Brit. Med. Jour.*, Aug. 5, 1876.

† Loc. cit., Case II.

on nerves, and Gluck, in 1882, at the Congress of German Surgeons, reported some astonishing successes in neuroplasty by transplantation. While the writer has not as yet performed this operation, he would not hesitate to make the experiment of transplanting nerve from some animal into a patient, did a suitable case present.

It may be well to add, no case yet known has had neuritis or tetanus, or have any untoward consequences resulted from this sort of surgery of the nerves. The operation deserves much more cordial recognition and acceptance than it has as yet received.

II. *Elongation of Nerves.*

Upon this portion of the paper, the writer dwelt considerably upon the literature, and many were the authorities quoted. Its application has apparently, therefore, not been restricted to neuralgia and contractures, but has been extended to anæsthesia and paralysis, great atrophy, convulsive affections, to tetanus, locomotor ataxia, and even to anæsthetic leprosy, etc.

To Nussbaum (says the writer) we owe a debt of gratitude for making the way clear and first putting "nerve-stretching" into practice. While resecting an elbow in 1860, his assistant accidentally but forcibly stretched the ulnar nerve, and it was noticed the tetanic cramp, with which the arm was previously affected, totally disappeared, which made a strong impression on his mind. In 1869, it happened that Bilroth had a patient who had received a violent contusion of the buttock, and subsequently developed epileptiform paroxysms. Under the impression that there was nerve irritation from some bone splinter, he denuded the nerve without finding anything wrong, but the nerve had been disturbed and more or less stretched, and recovery followed. Acting on these hints, Nussbaum for the first time practiced intentional elongation in 1872, the case being one of contracture of the upper extremity, consecutive to contusion. Recovery followed the operation. Without discussing the general merits of the operation, or the statistics, or extent of stretching that nerves will bear, nor yet the changes resulting therefrom other than clinical results obtained, we will be content in making synopsis of the five cases reported by the writer, that have not heretofore been placed on record, in three of which elongation was made on purely empirical grounds, and merely as physiological experiments.

CASE I. *Chronic Sciatica—Elongation—Recovery.*—W. L., aged 25, has suffered severely from sciatica for some five months; has lost considerable flesh; has been all this time under medical care, and for the last six weeks in the County Hospital, Chicago. A most varied internal treatment has been resorted to, as well as the use of electricity, part of the time intelligently applied. Patient was admitted into the Michael Reese Hospital, Chicago, and on June 15, 1883, he was anæsthetized, and the sciatic nerve of the affected side was stretched. The operation was made under the spray. By the 20th, when the dressings were changed. the wound was found to have healed per primam; pain was relieved at once, and at this date sensation and motion were found to be nearly normal. There was now and then a twinge during the next month.

July 20, when he was discharged, he simply noticed an occasional pain in one small spot in the calf of the leg.

September 1, he called to say he was perfectly well.

CASE II. *Toy Pistol Tetanus—Elongation of Brachial Plexus—Amelioration of Symptoms.*—A. G., aged 10. On the 4th of July, 1883, patient injured his left hand with a toy-pistol. On the 11th, at 4 A. M., he developed the first symptoms of tetanus. He was at once brought to the Michael Reese Hospital. Saw him at 11:30 A. M.; at that time there was moderate clonic spasm of the muscles of back of neck, with tonic exacerbations; found a suppurating wound in palm of left hand. The patient was anæsthetized; operated under the spray. The palmar wound opened, I found it foul and containing a piece of wadding, which was removed, and the cavity scraped. An incision along the inner border of the biceps brachii was made, the nerves isolated and stretched in each direction, including the median, ulnar, and internal cutaneous nerves, and that of Wrisberg. Wound was closed over a small drainage tube, and iodoform dressings applied. The patient improved decidedly after this procedure. Squibbs' ext. physostig. gr. 1-15 hypodermically every two hours; also sodii bromid. gr. x. every two hours, and chloroform inhalations during any tetanic seizures. Evening condition very satisfactory. Next morning about 7 o'clock he complained of pain about the heart, and died within an hour, apparently from spasm of the diaphragm. Autopsy not allowed.

CASE III. *Motor Paralysis of Right Leg, following Spinal Meningitis—Elongation of Sciatic Nerve—Negative Result.* A. K., aged 40, in July, 1881, was seriously ill, probably a spinal meningitis. At present he suffers from mono-plegia, the right lower extremity being the part involved. Has been under the care of several physicians, and recently a patient in the medical department of the Michael Reese Hospital, where, along with electricity, various other remedies were tried. Elongation of the sciatic as an experiment was proposed; patient accepted it *as such.* September 15, 1882, operation under the spray. The limb was suspended by the nerve, and stretched from above downward, and *vice versa.* Heart's action momentarily disturbed. Union *per primam.*

October 17, patient acknowledged perceptible improvement. Very little further improvement was made in several weeks longer that he remained in the hospital.

CASES IV. AND V. *Paræsthesia and Dysæsthesia of Unknown Origin—Elongation of Sciatic, and Later of Crural Nerves—No Result.* H. K., aged 32, has suffered fourteen years from perverted sensations, causalgia, etc., referred to integument of lower extremities, especially the right one. Of late complains of a feeling of intense cold, referred particularly to the area of distribution of the right sciatic and peroneal nerves. His intelligence is not of high order. Patient has been under the observation of Dr. Ernst Schmidt for years, who finally suggested *elongation* as a pure experiment, and admitted him to the Michael Reese Hospital, June 22, 1883. Sciatic and pero neal nerves were stretched, with the usual precautions. July 1, no improvement, and at patient's urgent request the anterior crural was stretched, and yet no benefit seemed to accrue. Each time the nerve was stretched downward in this case, and also an irregularity of the heart's action occurred for a few seconds.

*Commentary.* Case I exhibits the happy effects to be obtained in obstinate cases of sciatica resisting medication. The operation has a wide sphere of usefulness in this form of complaint, of which not a few cases are already on record.

Case II exhibits at least the temporary relief to be obtained by this measure in cases of tetanus. That we cannot afford to reject this method of treatment, for amelioration of symptoms is better than no impression at all, although in this case an error may have been committed in omitting also to stretch the musculo-spiral nerve; possibly too much sedative treatment was given the patient, and not sufficient stimulating nourishment; a not infrequent mistake.

Cases III, IV, and V were undertaken, as already remarked, on *purely experimental grounds,* and the patients accepted it as such; they further go to prove that we usually learn more by our failures than from our successes.

III—*Exploration of the Brain by the Hollow Needle.*

There is no *special literature* on this subject. To illustrate the ease with which it may be done, and its harmlessness, the author cited the following cases:

*Case I—Gunshot Wound of Brain—Trephining—Repeated Exploration for Pus—Extrusion of Fragment of Bone—Recovery.* J. L., age 20, on the afternoon of July 4, 1883, received a bullet-wound of the left parietal eminence; brought to the Michael Reese Hospital. The house-surgeon abstained from exploring the wound, but clipped the hair short and made antiseptic occlusion with iodoform cotton, also applied ice-bags. At evening there was complete right hemiplegia; pulse 90; pupils normal; patient conscious and rational; continued the ice-cap and gave directions to use catheter.

Next morning learned of patient having two convulsions during the night; he also lost consciousness; pupils normal, and respiration natural.

Exploration under the spray revealed a comminuted gunshot fracture; trephined; removed several small fragments, some of which were partially imbedded in the brain; removed also a piece of the small pistol bullet. Compression was then made and dressings of napthaline gauze applied. For several days patient's condition was satisfactory, except that the hemiplegia persisted.

July 13—Answered questions rationally.

July 18—There was free discharge of pus.

July 20—Symptoms of cerebral irritation; fear of abscess forming; dressings removed; introduced a well-cleansed needle of a hypodermic syringe through the cicatricial tissue, explored in different directions by its full length, never completely withdrawing the needle point. No pus, and but a few drops of blood entered the barrel of the syringe. No anæsthetic was given, nor pain complained of; no unpleasant effect of any kind followed.

July 23—Has some control of right leg, but the arm was in a contracted condition.

July 26—Patient very fretful and irritable; discovered a hard substance under the integument; upon cutting down (without an anæsthetic), removed a piece of bone of the size of a silver half dime, and the full thickness of the skull. To be certain that no abscess was forming, exploration was again made with the hypodermic needle as before, and again with negative results.

July 28—Patient discharged, wearing a lead plate to repress a tendency to hernia cerebri.

August 3—Patient steadily improving and regaining the use of his arm.

*Case II—Suspected Abscess of the Brain—Exploration.* This case presents a humorous side. H. H., age 38; admitted to the Buffalo General Hospital during the first week in January of the present year. No accurate history elicited; patient had facial paralysis of right side of the face; gave a very indefinite history of suppurative trouble of right middle ear dating back for years. Had also further paretic symptoms in the limbs; incoordination of ordinary movements; incontinence of urine and feces; pupils unequal, but sensitive to light; pulse and temperature normal. He gave *no* history of convulsive seizures of any kind; syphilis denied.

January 7—Was summoned to a consultation concerning the case; others preent, Drs. Wyckoff, Rochester and Gay. For several hours patient had been comatose, pulse had been as low as 48, and his general appearance was as of one suffering from compression of the brain. Bearing in mind the trouble in his middle ear, and the other part of the history of the case, an abscess of the right side of the brain, or a collection of pus in that neighborhood was suspected, and exploration was resorted to over the mastoid region, first trephining high enough to escape the lateral sinus. The longest needle of a hypodermic was then used, passing it inward, upward, downward, backward and forward, getting thereby only a few drops of blood. Wound was irrigated with sublimate solution, closed over a drainage tube, and a napthaline dressing applied; spray not used; very little ether required. The succeeding day, January 8, patient perfectly conscious. In short, this had been an epileptic seizure.

Janury 16—Wound perfectly healed over where the button of bone had been removed, and his general condition was improved.

February 5—Patient went home perceptibly better than when he entered.

Evidently here was an error in diagnosis, but an excusable one, as the man had not been in the hospital long enough to make a diagnosis possible, and where no history was obtainable, especially regarding his previous seizures. It is, moreover, obvious that the epilepsy in his case is secondary to some serious organic change, and a case of this kind, with its *post-mortem* revelations, was still fresh in the surgeon's mind, which possibly also influenced him unduly to operate. In spite of error, however, the patient was benefited rather than injured by the operation. Exploration of the brain, at least of its upper and enveloping portions by the hollow needle, is harmless, and it should find a recognized place both in our surgical practice and in our textbooks.

The paper evoked considerable discussion, as may be seen from the following remarks:

In discussion, Dr. J. G. Kiernan said Johnson (a Norwegian physician) had experimented on suture of nerves in fowls, and reported that to do so by the direct method, it required 45 days before the animal regained the normal function of the affected part, and 60 days when it was performed by the indirect method.

Regarding the second part of the paper—nerve-stretching—there is not much benefit to be derived from the operation, except in cases of neuralgia. The operation is not unattended with danger. During 1882, of 25 cases of locomotor ataxy reported where nerve-stretching was performed, five cases died from the operation. The same year, Graeme Hammond reported a case where the operation aggravated the disease. In a case of multiple cerebral sclerosis in which he was personally cognizant of the facts in the case, he reported the following result: The patient (a man) having previously been treated for a long time, visited an Eastern city and returned markedly improved. Upon inquiry, I was told by him that the posterior cord of the brachial plexus had been stretched. I examined the surface over the region of the clavicle, and at once saw that only the integument had been cut through. Now, here is a case of *imaginary improvement*, and the man really believes himself improved because, as he supposed and believed, the *real* operation had been done to relieve him. Regarding the case of tetanus reported in the paper, he did not think nerve-stretching was warranted after the source of irritation had been removed, the wound cleansed and dressed antiseptically, and the patient treated with physostigma. Regarding exploration of the brain, the speaker thought it an unjustifiable procedure, unless based on symptoms localizing the lesion in some portion of the brain.

Dr. J. J. M. Angear arose and debated the subject quite extensively. Regarding the vicarious action of nerves that the author alludes to, and their anastomoses, he did not fully understand. For it is a fundamental principle that nerves are continuous, and thought these words should not apply here, as we do to the circulatory system. Regarding the cases of suture of nerves of long standing the author speaks about, the speaker said, when a nerve is severed and does not at once heal, that nerve, or portion of it passes into fatty degeneration, and it is then no longer a nerve, and we might as well talk of restoring arteries after ligating them as of restoring nerve function after this manner.

To illustrate—We may have a painful stump after an amputation, and why? Because the ends of nerves supplying the parts have not passed through this degenerative process, but instead, little fibrous cords, or bulbs, or a tumor has formed, and oftentimes may require to be removed to relieve the increased sensibility. We do not have that immediate physiological action from suturing nerves—it takes weeks or many months before physiological action is established. With reference to elongation of nerves, great damage may be done in this procedure when this operation is performed. A surgeon (he doubts) never looks into the details or minutiæ, to see further about the nerve to be stretched, what is wrong, but proceeds to the stretching of it, and I very much doubt if peaceful, harmonious nerve action follows this operation, or at least in but one or two exceptions.

Exploration of the Brain.—This we cannot endorse as a society. If we operate on the cerebrum of a patient, fatal harm may result. I cannot see how it is the "harmless operation" the surgeon states it to be, and I think death more often occurs than beneficial results follow such procedures. Certainly there is great danger in probing the human brain, for a minute clot may form from a little hæmorrhage that may ensue.

Dr. G. C. Paoli: Let us admit, for the sake of argument, that suture of nerves and nerve-stretching will relieve suffering humanity. But no statistics that he knows of are yet published as to the percentage of successes. He does not doubt but that in a very few cases nerve-stretching may prove beneficial. We often admire the boldness of a surgeon, but what a surgeon does to-day he would, years ago, have been branded for as committing murder. Some of our colleagues who are old in the practice of their profession are, for like reasons perhaps, called "old fogies" because we do not go ahead and cut, but nature heals our cases and before long it may be that the operation of nerve-stretching will be performed in acute neuralgia, as it is now done to relieve the chronic forms of it. The operation for sciatica has been in vogue during the past ten or fifteen years, and does not always afford permanent relief.

Dr. D. A. K. Steele thought the discussion thus far had been quite severe in criticising the paper, and he thought immediate nerve suture to be justifiable; it should be done often where only the integument is sutured. Nerve stretching, he thought, is useless in tetanus and locomotor ataxy, whereas in obstinate sciatica it cures, and is a justifiable operation. The last portion of the paper on "Exploration of the Brain," is as yet certainly a mooted question. It seems to me that it can not be done without a blood clot forming or wounding a blood vessel, or that paralysis may ensue; if such is the case, then it is not a justifiable opertion. Some portions of the paper merit much praise, and as such I have been much interested.

Dr. R. Tilley thought the surgeon was not justified in doing more at the first consultation in the case of the toy-pistol tetanus cited in the paper, than the removal of the piece of wadding and the mere cleansing the wound and dressing it properly and using antiseptic measures, etc. Regarding suture of nerves and union of nerve tissues, experiments have been tried where crossing the nerves has been done by crossing the distal end over the central portion (X thus), and then it took from two to ten weeks before they became adherent and function restored; complete paralysis has occasionally resulted from the procedure.

A motion that the society do now adjourn prevailed.

